# Mental illness research in the Gulf Cooperation Council: a scoping review

**DOI:** 10.1186/s12961-016-0123-2

**Published:** 2016-08-04

**Authors:** Jason E. Hickey, Steven Pryjmachuk, Heather Waterman

**Affiliations:** 1 University of Calgary Qatar, P.O. Box 23133, Doha, Qatar; 2 University of Manchester, Oxford Rd, Manchester, M13 9PL United Kingdom; 3 Cardiff University, Cardiff, CF10 3XQ United Kingdom

**Keywords:** Arabs, Mental disorders, Mental health, Mental health services, Middle East, Qatar, Scoping review

## Abstract

Rapid growth and development in recent decades has seen mental health and mental illness emerge as priority health concerns for the Gulf Cooperation Council (Bahrain, Kuwait, Oman, Qatar, Saudi Arabia, and the United Arab Emirates). As a result, mental health services in the region are being redefined and expanded. However, there is a paucity of local research to guide ongoing service development. Local research is important because service users’ experience of mental illness and mental health services are linked to their sociocultural context. In order for service development to be most effective, there is a need for increased understanding of the people who use these services.

This article aims to review and synthesize mental health research from the Gulf Cooperation Council. It also seeks to identify gaps in the literature and suggest directions for future research. A scoping framework was used to conduct this review. To identify studies, database searches were undertaken, regional journals were hand-searched, and reference lists of included articles were examined. Empirical studies undertaken in the Gulf Cooperation Council that reported mental health service users’ experience of mental illness were included. Framework analysis was used to synthesize results. Fifty-five studies met inclusion criteria and the following themes were identified: service preferences, illness (symptomology, perceived cause, impact), and recovery (traditional healing, family support, religion). Gaps included contradictory findings related to the supportive role of the Arabic extended family and religion, under-representation of women in study samples, and limited attention on illness management outside of the hospital setting.

From this review, it is clear that the sociocultural context in the region is linked to service users’ experience of mental illness. Future research that aims to fill the identified gaps and develop and test culturally appropriate interventions will aid practice and policy development in the region.

## Background

The Gulf Cooperation Council (GCC) is a union of six Arabic states in the Persian Gulf, including Bahrain, Kuwait, Oman, Qatar, Saudi Arabia and the United Arab Emirates (UAE). These countries share many cultural, political, religious, economic, and geographical similarities [1]. The aim of the union is to promote regional development through coordination, cooperation and integration among member countries [2].

The GCC has benefited substantially in recent decades from large reserves of oil and natural gas. Qatar, for example, is a small, peninsular country that was once mainly utilized by coastal fishermen, pearl divers and nomadic Bedouin tribes [3]. Now, its capital city, Doha, is being transformed into an ultra-modern metropolis and the country has the highest GDP per capita in the world [4]. The UAE provides another example of rapid economic development; in less than 10 years it went from being one of the least developed countries in the world to a modern industrialized nation [5].

Islam is the foundation of cultural and social customs in the GCC [6]. It infuses nearly all aspects of life, from architecture, food choice, daily routine, social interactions, education, healthcare and more. Tolerance, hospitality and modesty are highly valued [7]. However, social customs also result in strict guidelines for what is considered appropriate behaviour in certain circumstances. For example, in Saudi Arabia, it is illegal to publically practice any religion other than Islam, and women are required to wear a gown (i.e. Abaya) and headscarf in public [8]. In other Gulf countries, these rules are often relaxed for foreigners. However, social and cultural norms sometimes clash with ongoing modernization and the influx of foreign workers. Because of this, some feel that traditional values are being threatened [9].

The recent and rapid changes in the region and the pressures or strains that have been mentioned above have seen mental health and mental illness emerge as priority health concerns for all countries in the GCC. In Kuwait, mental health was identified as one of six strategic priorities through a consultation with WHO [10]. As part of this agenda, mental health services are being integrated into primary healthcare, and community and home-based services are being developed. Oman also acknowledges the need to scale-up mental health services, particularly by increasing the number of available beds, providing training for primary care workers, and implementing a school health program [11]. In Qatar, a National Mental Health Strategy was recently developed [12]. This strategy focuses on system-wide change to reduce stigma, improve treatment seeking, increase availability of resources, scale-up the workforce, provide services in a variety of locations, and develop standards and guidelines. Bahrain, Saudi Arabia and the UAE have also emphasized mental health as a national priority and service development is underway in these counties as well [13–15].

Research on mental illness from GCC countries suggests that sociocultural factors influence people’s experience of mental illness in the region. For example, causal attribution of mental illness to demons (Jinn) prompt people to seek traditional or religious healers frequently [16]; shame can cause families to impose social isolation on a sick family member [17]; extended family structures can promote increased levels of family support and housing [16]; and religious (Islamic) influences have been linked to non-Western presentations of illness [18]. Hence, for service planning to be most effective, it cannot necessarily rely on international best practices and evidence from other countries. However, it is widely acknowledged that there is limited local research available to guide contextually-appropriate development of mental health services in the region [11, 12].

The current article aims to systematically review and synthesize regional literature that reports service user perspectives on mental illness in GCC countries, identify major gaps in the literature, and suggest directions for future research. This information will facilitate the development of mental health services in the GCC. It will also provide information for mental health practitioners in non-GCC countries who provide services for Arabic people.

### Theoretical framework

Arksey and O’Malley’s [19] framework was used to develop the review protocol. According to one of the most commonly cited definitions, a scoping study “*aims to map rapidly the key concepts underpinning a research areas and the main sources and types of evidence available…*” ([20] as cited by [19], emphasis in original). Scoping studies tend to be inclusive of a range of research designs regardless of where the research sits on the ‘evidence hierarchy’ [21, 22] and seek to provide greater conceptual clarity [23].

Arksey and O’Malley [19] identify four possible reasons to conduct a scoping study: to examine the extent, range and nature of research activity; to determine the value of undertaking a full systematic review; to summarize and disseminate research findings; and to identify research gaps in the existing literature. Most reports on scoping studies tend to incorporate a combination of these objectives, and outcomes typically include identification of themes in the literature, gaps that have yet to be addressed, and tangible recommendations for practice and research [24–27]. These characteristics make a scoping study well-suited for the aims of the current review.

## Methods

### Inclusion criteria

Articles covering one or more of the following common or clinically relevant illnesses [28, 29] were included: mood disorders, alcohol and substance use disorders, schizophrenia, Alzheimer’s and other dementias, anxiety disorders, obsessive compulsive disorder (OCD), personality disorders, and phobias. Additionally, studies had to be empirical (i.e. based on observed and measured phenomena and deriving knowledge from actual experience rather than from theory or belief), published in English, and conducted in the GCC (Qatar, Saudi Arabia, Kuwait, Bahrain, the UAE, Oman). Only studies that reported subjective data from participants were included because the perspectives and lived experiences of service users are critical for informing a recovery-oriented understanding of mental illness. Subjective data was defined as opinions or experiences collected directly from participants. Articles not meeting these criteria were excluded from the review.

### Identifying relevant studies

First, several databases (CINAHL, Anthropology Plus, MEDLINE, SocINDEX, PsycINFO, Embase, and NCBI PubMed) were searched. The following search string was developed with the assistance of a librarian: ((MM “Mental Health”) OR (MM “Mental Disorders +”) OR (MM “Mentally Ill Persons”)) AND (Cooperation Council for Arab states of the Gulf OR CCASG OR Gulf Cooperation Council OR GCC OR Qatar* OR Emirat* OR Abu Dhabi* OR Bahrain* OR Kuwait* OR Saudi Arabia* OR Oman*). Second, several regional journals (*Arab Journal of Psychiatry*, *Eastern Mediterranean Health Journal*, *Avicenna*, Journal of *Local and Global Health Science*, *Journal of Local and Global Health Perspectives*, *QScience Connect*, and *Qatar Medical Journal*) were hand searched. Databases and regional journals were searched from inception to December 2013. Finally, reference lists of articles identified in the previous two strategies were searched.

### Study screening

**Fig. 1:**
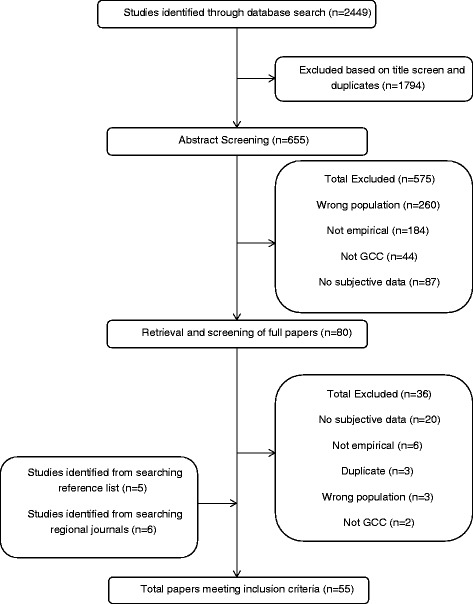
Strategy used to identify studies

Figure 1 illustrates the study identification and screening process. The first author screened titles from the initial database results (*n* = 2449) and removed duplicates and irrelevant articles. Inclusion criteria were then applied to full abstracts for the remaining articles (*n* = 655) by the first author. The other two authors (SP, HW) each screened a 5% random sample of abstracts to ensure consistent application of inclusion criteria. Full papers for the articles that passed abstract screening (*n* = 80) were obtained and read in full by two authors. An additional 36 articles were excluded at this stage. Eleven articles were included from the hand search of regional journals and reference lists. Fifty-five articles were included in the final data analysis. Table 1 demonstrates how each of these 55 studies met the inclusion criteria.

**Table 1:** Inclusion data for all articles included in final review

Author (date)	Design	Country	Diagnosis	Subjective data
Al-Faraj & Al-Ansari (2002) [58]	Cross-sectional	Bahrain	Schizophrenia	Perceived cause of symptoms/illness
Daradkeh & Moselhy (2011) [39]	Cross-sectional	Bahrain	Death anxiety (thanatophobia)	Degree of death anxiety and focus of anxiety
Derbas & al-Haddad (2001) [80]	Cross-sectional	Bahrain	Substance abuse	Factors associated with relapse
Shooka et al. (1998) [50]	Cross-sectional	Bahrain	Obsessive compulsive disorder	Characteristics of obsessions and compulsions
Suleiman et al. (2002) [45]	Cross-sectional	Bahrain	Depression	Symptomology
Al-Ansari et al. (1989) [51]	Cross-sectional	Kuwait	Schizophrenia	Characteristics of hallucinations and delusions
Al-Ansari & Negrete (1990) [36]	Comparative cross-sectional	Kuwait	Alcohol abuse/dependence	Perception drinking patterns and associated impacts
Al-Kandari et al. (2001) [81]	Cross-sectional	Kuwait	Substance dependence	Reasons for initiating drug use
Al-Kandari et al. (2007) [37]	Cross-sectional	Kuwait	Substance dependence	Preferences for illicit drugs, craving/withdrawal patterns, related problems
Al-Saffar et al. (2008) [82]	RCT	Kuwait	Depression	Satisfaction with educational intervention, sources of medication information
Bilal (1988) [83]	Cross-sectional	Kuwait	Substance abuse	Perceived problems
Bilal et al. (1987) [64]	Cross-sectional	Kuwait	Alcoholism	Reflections on illness
Bilal et al. (1987) [64]	Cross-sectional longitudinal	Kuwait	Alcohol dependence	Problems associated with drinking
Chaleby (1985) [57]	Cross-sectional, chart review	Kuwait	Mixed^a^	Perceptions that marriage was related to psychiatric disorder
Demerdash et al. (1981) [66]	Comparative cross-sectional	Kuwait	Substance abuse	Reasons for drinking, reasons for combining substances
El-Islam et al. (1988) [84]	Cross-sectional	Kuwait	Depression	Timing of onset and symptomology of hypochondriasis
El-Islam et al. (1988) [43]	Cross-sectional	Kuwait	Depression	Core depressive symptoms
Suleiman et al. (1989) [85]	Comparative cross-sectional	Kuwait	Mixed^a^	Reported negative life events
Suleiman et al. (1986) [47]	Cross-sectional	Kuwait	Mixed^a^	Provoking factors for attempted suicide
Zahid et al. (2010) [86]	Cross-sectional	Kuwait	Schizophrenia	Satisfaction across nine life domains
Zahid & Ohaeri (2010) [53]	Cross-sectional	Kuwait	Schizophrenia	Perceived caregiving burden
Zahid & Ohaeri (2010) [87]	Qualitative interviews	Kuwait	Schizophrenia	Presence of hallucinations, delusions and negative symptoms
Zahid & Ohaeri (2013) [88]	Cross-sectional	Kuwait	Schizophrenia	Met/unmet needs
Zahid et al. (2010) [89]	Cross-sectional	Kuwait	Schizophrenia	Satisfaction with mental health services
Zaidan et al. (2006) [42]	Cross-sectional	Oman	Alcohol abuse	Impact of alcohol abuse
Bener et al. (2013) [90]	Cross-sectional	Qatar	Mixed^a^	Reasons for non-compliance
Bener & Ghuloum (2013) [91]	Cross-sectional	Qatar	Mixed^a^	Satisfaction with psychiatric consultation, important topics to include in consultation
El-Islam (1982) [63]	Mixed	Qatar	Schizophrenia	Family caregiving roles
El-Islam (1994) [48]	Cross-sectional	Qatar	Mixed^a^	Phenomenology of various phobias, perceived cause of illness
Ghuloum et al. (2010) [92]	Prospective cross-sectional	Qatar	Mixed^a^	Satisfaction with psychiatric consultation
Kent & Wahass (1996) [52]	Comparative cross-sectional	Saudi Arabia	Schizophrenia	Characteristics and content of hallucinations
Chaleby (1986) [93]	Retrospective	Saudi Arabia	Alcohol/substance abuse	Frequency of stressors
Chaleby & Raslan (1990) [94]	Cross-sectional	Saudi Arabia	Social phobia	Perceptions of childhood, parents, work environment
Mahgoub & Abdel-Hafeiz (1991) [49]	Cross-sectional	Saudi Arabia	Obsessive compulsive disorder	Characteristics of obsessions and compulsions
Shahin & Daly (1999) [95]	Cross-sectional	Saudi Arabia	Mixed^a^	Knowledge, attitudes and beliefs about psychiatric medications
Abalkhail (2001) [35]	Prospective, comparison group	Saudi Arabia	Substance dependence	Symptoms experienced during detox
Al Sughayir (2000) [96]	Case-control	Saudi Arabia	Mixed^a^	Possession beliefs
Al-Habeeb et al. (2013) [43]	Cross-sectional	Saudi Arabia	Depression	Components of suicidal ideation, frequency/duration of suicidal ideation, control over suicidal thoughts, reasons and deterrents for attempting suicide
Al-Habeeb & Qureshi (2000) [56]	Cross-sectional	Saudi Arabia	Mixed^a^	Reasons for smoking, reasons for not smoking, associated impacts, experiences with smoking cessation
Al-Nahedh (1999) [65]	Cross-sectional	Saudi Arabia	Substance abuse	Reasons for initiating drug use
Alshowkan et al. (2013) [97]	Cross-sectional	Saudi Arabia	Schizophrenia	Perceived quality of life
Al-Solaim & Loewenthal (2011) [34]	Qualitative interviews	Saudi Arabia	Obsessive compulsive disorder	Encounters with traditional healers, supernatural beliefs, impact of religion, impact of illness
Al-Subaie (1994) [61]	Cross-sectional	Saudi Arabia	Mixed^a^	Perceived outcome of traditional healing
Chaleby (1988) [98]	Cross-sectional	Saudi Arabia	Mixed^a^	Factors perceived to be associated with marital discord
Daradkeh & Al Habeeb (2005) [99]	Cross-sectional	Saudi Arabia	Schizophrenia	Perceived level of health and quality of life
Iqbal (2002) [40]	Cross-sectional	Saudi Arabia	Substance abuse	Characteristics of hearing changes after amphetamine use
Qureshi (1992) [41]	Cross-sectional	Saudi Arabia	Substance abuse	Reasons for using, symptoms associated with craving, methods to obtain drug
Qureshi et al. (1998) [62]	Cross-sectional	Saudi Arabia	Mixed^a^	Reasons for seeking, and perceived outcomes of, traditional healing
Zarrouk (1975) [54]	Cross-sectional	Saudi Arabia	Schizophrenia	Characteristics of hallucinations
Zarrouk (1978) [55]	Cross-sectional	Saudi Arabia	Schizophrenia	Characteristics of hallucinations and delusions
Al Zarrad & Abu-Mugaiseeb (2002) [100]	Prospective	UAE	Mixed^a^	Attitudes and satisfaction towards service
Amin & Hamdi (1995) [32]	Retrospective chart review, prospective follow-up	UAE	Mixed^a^	Symptomology
Daradkeh & Karim (1994) [60]	Cross-sectional	UAE	Schizophrenia	Negative impacts of illness
Hamdi et al. (1997) [44]	Cross-sectional	UAE	Depression	Characteristics of depressive symptomology
Salem et al. (2009) [33]	Cross-sectional	UAE	Mixed^a^	Outcomes after consultation with traditional healer, perceived cause of illness

^a^Includes one or more targeted disorders

### Data extraction

The first author (JH) extracted data from all 55 articles using a structured extraction template. This template was developed and piloted by the review team. Two authors (SP, HW) each extracted data from half of the final set, meaning data was independently extracted from each article twice. The two extractions were compared and discrepancies resolved through group discussions between the three authors.

Data were extracted from each study under the following categories: general information (e.g. year, profession of primary author), methodology (e.g. design, study location), sample characteristics (e.g. gender, diagnosis), results (e.g. main outcome, subjective outcome), and discussion (e.g. limitations, conclusions). Strengths and weaknesses of individual studies were assessed and recorded during the extraction process.

### Data analysis

Descriptive statistics were compiled to illustrate the “*extent, nature and distribution*” [19] of the literature identified. A thematic analysis of the subjective data was also conducted. The purpose of this analysis was to identify and elaborate on the main concepts addressed by the literature. One of the main criticisms of scoping studies is that there is a lack of transparency and rigour in synthesizing and presenting thematic results. Framework analysis [30] was chosen as an analytic approach in order to address these issues.

Framework analysis proceeds through a series of logical steps to reach a narrative summary of the results. Data extraction forms were read and re-reread to increase familiarity, then codes were applied to identify key concepts within the data. Once all extraction forms had been coded, the initial codes were reviewed and revised to create a conceptual framework. The conceptual framework was then applied back to the extraction form to assess its fit. When the extraction forms had been recoded according to the conceptual framework, the data was entered into a matrix where results could be examined across themes (columns) or article (rows). The majority of analysis was conducted by one author (JH). However, initial codes and a draft of the conceptual framework was reviewed and discussed by the entire team, and the matrix was reviewed to ensure logic and consistency.

## Results

**Fig. 2:**
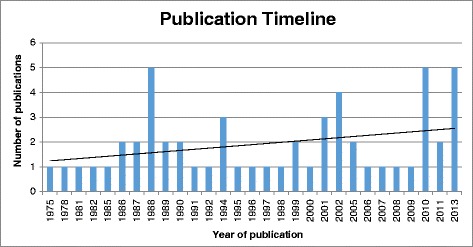
Number of publications by year of publication

Figure 2 displays the publication timeline. The oldest included study was published in 1975 and there are three notable peaks in publication frequency in 1988, 2001–2002, and 2010–2013. There is an upward trend in publications over the entire period.

The greatest number of studies were conducted in Saudi Arabia (*n* = 21, 38%) followed by Kuwait (*n* = 18, 33%). Five studies (9%) were conducted in each of Qatar, Bahrain and the UAE. One study (2%) was conducted in Oman.

Psychiatrists acted as first author on the majority of publications (*n* = 39, 70%) followed by researchers from medicine (specialty unspecified) and nursing, who authored four papers each (7%). Professions of other first authors included epidemiology (*n* = 2, 4%), psychology (*n* = 2, 4%), the behavioural sciences (*n* = 1, 2%), and pharmacy (*n* = 1, 2%). The profession of the primary author was unspecified in two cases.

**Fig. 3:**
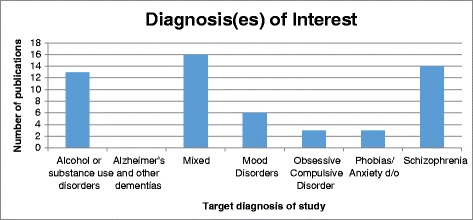
Number of publications by target diagnosis

Figure 3 displays which diagnoses were investigated. The most common target was a mixed sample (*n* = 16, 28%), comprising individuals with a range of diagnoses. This was followed by studies examining exclusively schizophrenia (*n* = 14, 25%) and alcohol or substance use disorders (*n* = 13, 23%). None of the included articles targeted Alzheimer’s or other dementias.

Table 2 displays sample size by gender and diagnosis. Overall, males outnumber females by a ratio of 2.2 to 1. The largest gender imbalance occurred for alcohol or substance use disorders where males outnumbered females by a ratio of 72.6 to 1. The only disorder where females were over represented was OCD, where females outnumbered males by a ratio of 2.6 to 1.

**Table 2:** Gender of participants for each of the studied diagnostic categories

Diagnosis of interest	Male	Female	Ratio
Alcohol or substance use disorders	1452	20	72.6 to 1
Depression	668	263	2.5 to 1
Obsessive compulsive disorder	27	70	1 to 2.6
Phobias/anxiety disorder	165	65	2.5 to 1
Schizophrenia	1111	413	2.7 to 1
Mixed sample	2704	1958	1.4 to 1
Total	6127	2789	2.2 to 1

The vast majority of studies (*n* = 42, 84%) recruited participants from public psychiatric treatment centres. Of these, half recruited from inpatient or detox units, while half recruited from outpatient departments. Two studies (4%) recruited from private clinics, and only one study (2%) recruited participants from the community. Twelve studies (24%) were unclear about recruitment location.

Several themes and sub-themes emerged during analysis, including service preferences, illness (perceived cause, symptomology, impact), and recovery (traditional healing, family support, religion). The following section, which critically synthesizes the results of this review, is organized according to these themes and sub-themes.

### Service preferences

Gender seemed to influence service preferences in several studies. For example, Bener and Ghuloum [31] found that patient gender affected the type of topics seen as most important when receiving treatment from a psychiatrist. In that study, males viewed discussions with a doctor about treatment options as being most important, while females prioritized explanations of the condition and the underlying cause. Surveyed preferences were limited to interactions with a psychiatrist; thus, it is not possible to determine preferences across other professional groups. Additionally, the results were collected using a brief questionnaire, which limited choices and did not explore the underlying reasons for observed differences. However, the study suggests that gender is an important consideration for service delivery in this region.

The importance of gender is reinforced by Amin and Hamdi [32], who found gender to have an influence on where participants preferred to seek treatment. The authors found that females in need of psychiatric care tend to present at the emergency department, while males were more often seen in the outpatient department. Amin and Hamdi [32] suggest several reasons for their observed difference but these were conjecture and not grounded in the results. Additionally, this study is now 20 years old and with the recent modernization in the region, results may no longer be accurate. Thus, there has been no recent research to examine service users’ preferences across a range of services within the healthcare system.

Other studies, however, have demonstrated a preference for services outside of the healthcare system. Salem et al. [33] found that nearly half of the sample went to a faith healer prior to seeking psychiatric care. The majority of these participants continued to see a faith healer even after engaging with psychiatric services. Because convenience sampling was used in this study it is difficult to generalize results to the wider population. However, the participants originated from various countries in the Gulf region, had a range of diagnoses, and included nearly equal proportions of men and women. This diverse sample adds to the generalizability of the study. The author concludes that mental health professionals need to be aware of patient preferences for traditional healing and understand the reasons why they sometimes refuse medical treatment.

A comparable study conducted by Al-Solaim and Loewenthal [34] in Saudi Arabia demonstrated that psychiatric services seem to be seen as a last resort when other options (e.g. faith healers) are not successful. This reinforces Salem et al.’s [33] argument that mental health professionals should not ignore the contribution of traditional healers to service users’ treatment. However, Al-Solaim and Loewenthal’s [34] results are drawn solely from the experiences of 15 women. It is possible that they may not represent the majority view. Men, in particular, as demonstrated earlier, may have different preferences.

### Illness: symptomology

Symptomology was one of the most commonly occurring themes in the included articles. Clinical presentation of mental illness was described for alcohol and substance abuse [35–42], depression [32, 43–45], suicide [46, 47], panic disorder [48], OCD [34, 49, 50], schizophrenia [51–55], smoking behaviour [56], and hypochondriasis [18]. Several of these articles emphasized the unique presentation of certain illness within the Arab context.

For example, Shooka et al. [50] demonstrated that religious and blasphemous thoughts were the most common obsessions in patients with OCD, while repeated prayer-related cleaning and washing was the most common compulsion. The authors suggest that there are higher levels of religious content in participants from a strict religious context. These findings were reinforced by Mahgoub and Abdel-Hafeiz [49], who found an Islamic focus for obsessions and compulsions in OCD. In particular, prayer and body washing were the most common obsessions and religious repeating and religious washing were the most common compulsions. Both these studies are quite old; however, the Islamic influence on symptomology was also apparent in Al-Solaim and Loewenthal’s [34] more recent study on OCD. An additional finding in this more recent study was that symptoms in the religious domain were the most disturbing for patients and their families. Despite their limitations, these studies suggest that the content and focus of OCD symptoms are culturally influenced.

Research on other disorders has also demonstrated a contextual influence. For example, a study by Kent and Wahass [52] on schizophrenia in Saudi Arabia found that hallucinations had more religious and superstitious content compared to a sample from the United Kingdom. Zarrouk [55] found that delusions also differed from non-Arabic samples, with Saudi patients more frequently believing they were being ‘made’ to do things. In a study on depression by Hamdi et al. [44], four main types of symptom variation (compared to non-Arabic studies) were identified, namely variations in idioms (e.g. heavy/tense vs. depressed/sad), use of somatic metaphors (e.g. ‘my body is shattered’), influence of religion (e.g. denying acts considered *haraam*/forbidden), and behavioural alterations (e.g. going into desert to stare at nothing). Several of these variations clearly reflect the local context.

Taken together, these articles demonstrate a contextual influence on symptomology in the GCC. It should be noted that religion is also associated with symptoms in non-Arabic contexts. However, the religious content of symptoms in this review are clearly shaped by the Islamic context. This finding has appeared repeatedly over a considerable period of time (1978–2011), which suggests that this is a somewhat stable phenomenon. Many of these authors reasonably suggest that an understanding of local variations in clinical presentation is important for accurate diagnosis and treatment of service users.

### Illness: perceived cause

The perceived cause of mental illness was often external. For example, participants with a diagnosis of nosophobia attributed the cause to over-investigation of minor complaints by physicians [48]. Similarly, over one-third of women in polygamous marriages attributed their illness to their marriage [57], the majority of caregivers attributed the cause of illness to social stressors [58], and those with thoughts of self-harm attributed these thoughts to the devil [48]. Taken individually, the design weaknesses (e.g. convenience sample, bias, cross-sectional design) in these articles prevent reliable conclusions from being made. However, the consistent external causal attribution across studies lends credibility to the findings.

One particular class of perceived external cause, the supernatural (e.g. black magic, jinn, evil eye), seems particularly common. For example, Salem et al. [33] found that about one-third of participants attributed their illness to supernatural factors, while one-third attributed them to psychiatric problems and one-third were unsure. Similarly, Al-Solaim and Loewenthal [34] and Al-Sughayir [59] found that the majority of patients attributed their illness to possession by jinn (i.e. evil spirits). This external attribution to supernatural forces can be protective as certain symptoms may not be as stigmatized and may not produce such strong feelings of guilt [34, 48]. In fact, those with the evil eye may see themselves as having some positive attribute worthy of envy, which boosts their self-esteem [34]. Again, there are design weaknesses (e.g. bias, unrepresentative samples), but the consistent findings on supernatural attribution warrant further, more rigorous investigation.

### Illness: perceived impact

Most of the data under this theme come from studies on alcohol and substance abuse, which limits applicability to people with other diagnoses. Additionally, there was a certain amount of heterogeneity in impact outcomes. For example, Al-Ansari and Negrete [36] surveyed people undergoing treatment for alcohol abuse. Participants felt guilty about drinking, that it caused their family/friends to worry about them, that it sometimes created interpersonal problems, and caused them to neglect personal responsibilities. In contrast, Zaidan et al. [42] found that the majority of their sample never felt guilty about their drinking. Based on the information presented, the samples in the two studies seem comparable, aside from country of residence. Hence, the reasons for the observed differences are unexplained. Additionally, both studies enrolled males only and therefore results are further limited.

Daradkeh and Karim [60] and Al-Solaim and Loewenthal [34] offer limited information on illness impact among people with schizophrenia and OCD, respectively. These included barriers to social inclusion (schizophrenia) and interpersonal problems with family members (schizophrenia and OCD). However, the small sample size used in these studies means that further investigation is needed before reliable conclusions can be made.

Another major limitation in the majority of the studies on alcohol and substance abuse is their almost exclusive reliance on questionnaires to collect data. This limits the depth and richness of information collected and prevents participants from sharing relevant information that is not covered on the questionnaire. Thus, despite the relatively large number of studies on this topic, understanding remains limited.

### Recovery: traditional healing

As mentioned above, a large proportion of psychiatric patients visit a traditional healer prior to seeking medical help [32–34, 58, 61]. This practice was perceived to contribute to recovery in a variety of ways. However, there were mixed perceptions on the effectiveness of traditional treatment.

Qureshi et al. [62] found that some participants with depressive or catatonic symptoms reported a temporary improvement from traditional treatment but that most were unsatisfied. Similarly, Salem et al. [33] found that about half of participants with a range of diagnoses experienced only a temporary benefit, with others experiencing no benefit at all. Both studies interpreted ‘benefit’ as a reduction in symptoms.

While most authors exploring the effectiveness of traditional healing seem to assume that symptom reduction is of primary importance, this may not necessarily be the case for service users. For example, Al-Subaie [61] found that, even those who did not perceive their symptoms to be reduced, reported feeling that God would reward them for having faith in traditional healing methods, which are primarily based on religious beliefs. This finding suggests that service users may place value on treatment benefits other than a reduction of symptoms. However, these other potential benefits are largely ignored in the studies reviewed, as is the relative importance of various benefits to participants.

A further limitation in the studies included in this section is selection bias. Since participants were selected from the population of people seeking psychiatric treatment, it is possible that others received longer lasting benefit from traditional treatments and did not subsequently access services. A broader sampling strategy (e.g. including people with mental illness who do not make regular use of psychiatric services) would be necessary for a more accurate investigation.

### Recovery: family support

The extended nature of the Gulf Arab family was frequently addressed as being a source of support for service users. Potential supportive roles of the family in one study included medication supervision, being tolerant of short periods of withdrawal, helping to find acceptable ways to describe and understand the illness, not expecting anything in return for their help, and assistance in filling leisure time [63]. While these caregiving themes make sense intuitively, the author introduces subjectivity and bias into the analysis and does not consider alternate or contradictory views. Additionally, the study was conducted over 20 years ago and no similar studies have been conducted to support or refute the author’s findings. It is also possible that the supportive role of families has changed in the ensuing period of rapid socioeconomic development.

Studies also suggested that support is more common in extended families compared to nuclear families and that those living in extended families had more social contact, better personal hygiene, less active symptoms, and better treatment outcomes [63, 64]. Conversely, the extended family was also perceived to have a negative impact; tension or stress within the family were cited as reasons for substance abuse [65] and attempted suicide [47], and were associated with higher disease severity [53]. Unfortunately, only the study by Zahid and Ohaeri [53] was recent enough to consider these influences within a modern context, and even this study did not examine the issue in depth. Hence, while it seems likely that the family plays a role in service users’ recovery from mental illness, the nature of this role in a modern context remains unclear.

### Recovery: religion

Committing acts that were incongruent with the teachings of Islam led to feelings of guilt and lower self-esteem [34, 48]. The more compliant a person was with the values of their faith, the more pride they felt [34]. However, compulsive religious acts relating to mental illness sometimes interfered with daily life and led to treatment seeking. Religion, however, particularly prayer, was still seen as one of the main ways to cope with mental illness and related stress. For example, being religious was associated with lower levels of death anxiety [39] and alcohol abuse [64, 66].

The majority of the studies included in this section incorporated religion as a minor variable that was a small part of a larger study. Thus, a systematic, in-depth investigation of religion has not been undertaken. Despite a lack of direct evidence, many of the reviewed studies argued that religion had an impact on certain aspects of participants’ illness, including symptomology (e.g. [43]), treatment seeking (e.g. [37]), etc. However, these claims and their underlying assumptions are currently unsubstantiated by the literature. In other words, broad assumptions were sometimes made, based solely on the authors’ own personal beliefs or professional understanding about the value of religion. Thus, while it seems likely that religion plays a role, it is difficult to objectively interpret the impact of religion based on the literature included in this review.

## Discussion

This systematic scoping review was undertaken in order to synthesize regional literature on service users’ experience of mental illness, identify gaps in the literature, and identify opportunities for future research; 55 articles were included in the review.

The included studies offer a small glimpse into service preferences, including a preference for initial consultations with faith healers. The use of faith healers to treat mental illness has also been documented in other developing countries. For example, a study conducted in rural India reported that faith healing is widely used and that many people seek traditional services before medical psychiatric services [67]. Services of faith healers are also commonly used in Ghana. This is because traditional healers offer more culturally appropriate models of understanding illness, higher levels of psychosocial support, and easier accessibility [68]. A study in Zimbabwe found that three quarters of people sought both traditional and medical treatment for mental illness; however, in this study, biomedical care providers were the most common point of first contact [69].

The frequent use of traditional healers has implications for government-run psychiatric services. The title of the article by Ae-Ngibise et al. [68] summarizes these implications quite well: ‘*Whether you like it or not, people with mental health problems are going to go to them* [faith healers]’. While some collaboration currently occurs in Qatar, ongoing service development efforts should aim to identify opportunities to incorporate safe, appropriate traditional healing as part of a comprehensive service.

External stressors, particularly the supernatural (i.e. jinn, black magic, evil eye) were frequently seen as the root cause of mental illness. This indicates a need to address cultural beliefs and social factors when treating mental illness. A recently published review found that psychiatric symptoms are commonly attributed to the supernatural among Muslims worldwide [70]. The authors claim that this external attribution has diagnostic and treatment implications. For example, biomedical treatment may not be accepted if underlying cultural beliefs about the supernatural are not addressed [71]. Therefore, practitioners should foster an awareness of traditional beliefs and be open to incorporating these as part of the therapeutic process.

Symptomology was described for most major mental illnesses and several similarities and differences were identified compared to typical presentations in the West. Variations in presentation of mental illness across cultures has been widely reported and debated in the literature [72]. Variations create difficulties in applying standard diagnostic criteria such as the DSM-5 or the ICD-10 in cultural contexts that differ from the West. This provides fuel for an argument that the dichotomous nature of diagnostic categories are unhelpful in the treatment of people with mental health issues [73, 74]. Unlike physical illness, mental illness is predominantly subjective (i.e. unmeasurable); interpretation and explanation of symptoms by the patient is influenced by sociocultural understanding [75, 76]. Attending to a person’s subjective experience of their illness and the overall impact of the illness on their life can lead to a more holistic understanding of the patient and better treatment outcomes [77]. It is worth noting that this practice aligns well with recovery-oriented care.

Other sociodemographic and sociocultural factors, such as family support, marriage, religion, education, financial status, gender, birth order, and nationality/ethnicity, were also investigated. However, the relationship of these factors to mental illness was difficult to assess due to limited data and weaknesses of the included studies. These areas provide fertile ground for future research.

The articles included in this review add important, contextually-relevant data to our understanding of mental illness in the region. However, despite nearly 40 years of research addressing the impact of sociocultural factors on people’s experiences of mental illness, a limited understanding of these issues remain. For example, the Arab extended family is widely acknowledged in having a key supportive role for those with mental illness. However, it has also been reported as a source of stress and conflict. Religion influences all aspects of life in Arab society and provides a source of strength and support for people with mental illness. However, forbidden acts, such as alcohol and substance abuse and suicide, still occur, and cause guilt and worry for patients and their families. Widespread belief in the influence of the supernatural on mental illness has been documented. These beliefs fall within the scope of modern mental health practice, but there is little evidence of how they can be incorporated successfully into a contextually-relevant model of psychiatric care and recovery. Research into the interplay between psychiatry and traditional healing is also lacking; service users place value on consultation with faith healers, but it is unclear how these services might be incorporated into a cohesive system of mental health service in the region, or even if they should be incorporated. The concept of stigma is conspicuously absent from the articles and little effort has gone into measuring or describing stigma towards mental illness in the GCC. Also absent from the literature are investigations into service users’ self-management of their illness. Most service users spend the majority of their lives outside of the healthcare system. Yet, virtually nothing is known about the strategies and resources they use to minimize the day-to-day challenges of mental illness.

### Strengths and limitations

This review is the first of its kind to be conducted in the Gulf region. Two other reviews of mental health research have been published [78, 79]. However, the scope of these reviews is limited to the frequency, distribution and topics of publications. This review is the first to synthesize the results of research on mental illness in this region. Additionally, the systematic approach undertaken for this review facilitated a relatively objective synthesis that was rigorously conducted. Finally, the use of Framework Analysis overcomes a major criticism of scoping studies by providing a transparent method of analysis where thematic results can be clearly linked to individual studies.

This review is limited by the general weaknesses in the body of literature. For example, most of the research included in this review seems to be unfunded. This implies that proposals may not have been subjected to peer review. While lack of funding does not necessarily imply low quality, many of the papers reviewed here would have benefitted from additional peer review during the planning stage. Increasing the funding available for mental health research could help to improve the overall quality of the research (e.g. through the scientific review process) and guide the focus of future research to ensure it is ethically sound and relevant for the development of practice and policy.

The evidence base consists primarily of cross-sectional studies aimed at developing foundational knowledge. This type of evidence does not allow for causal inferences to be made and only provides a snapshot of a phenomenon at one point in time. This means that the relationships between sociodemographic factors and illness experiences are still not well understood. Longitudinal research would help to increase understanding of illness trajectory outside of the hospital setting, while interventional research would help to improve the transformation of knowledge into practice. Additionally, the vast majority of included research was quantitative, meaning that results of the review lack depth. Incorporating more qualitative research has the potential to clarify important issues, help to develop a better understanding of service users’ perspectives, and build contextually relevant mental health theory that can be applied and tested through subsequent research. Qualitative research would also facilitate development of contextually valid measurement scales and questionnaires.

Underrepresentation of women in the studies highlights the need to look beyond convenience samples in psychiatric research at ways of identifying more representative samples. The gender imbalance implies that review findings are more relevant to the male psychiatric population. Finally, very few studies include family members or caregivers, even though most acknowledge the contribution of social support to recovery. Thus, understanding of caregivers’ potentially supportive role in the region remains limited.

## Conclusion

Despite the limitations of the reviewed literature, we can conclude that the sociocultural context in the Gulf region is linked to people’s experience of mental illness. However, service users’ perceptions and understandings about the nature of the context-illness experience relationship have not been systematically explored. This is particularly true for the process of self-management of illness outside the hospital setting. Interventions that are developed based on this limited understanding may have limited effectiveness and acceptability. While many questions can be derived from the identified knowledge gaps, two seem prominent:

1. What strategies do service users in Arabic countries use to self-manage their mental illness outside of the hospital setting?
2. What treatments and interventions would be most effective and acceptable to support this self-management?

Future research that attempts to address these two questions will increase the capacity of Arabic mental health services to provide efficient and culturally appropriate support to service users.
